# Relationship between DTI-MRI derived metrics and radiotherapy dose range in the contralateral cerebrum in lower grade glioma

**DOI:** 10.3389/fonc.2025.1406340

**Published:** 2025-07-09

**Authors:** Justyna Kłos, Hildebrand Dijkstra, Hiska L. van der Weide, Jan H. Potze, Peter F. Sinnige, Kelvin Ng Wei Siang, Rudi A. J. O. Dierckx, Ronald J. H. Borra, Miranda C. A. Kramer, Anouk van der Hoorn

**Affiliations:** ^1^ Department of Nuclear Medicine and Molecular Imaging, University of Groningen, University Medical Center Groningen, Groningen, Netherlands; ^2^ Department of Radiology, University of Groningen, University Medical Center Groningen, Groningen, Netherlands; ^3^ Department of Radiation Oncology, University of Groningen, University Medical Center Groningen, Groningen, Netherlands; ^4^ Department of Radiotherapy, Erasmus MC Cancer Institute, University Medical Center Rotterdam, Rotterdam, Netherlands; ^5^ Holland Proton Therapy Center, Department of Medical Physics & Informatics, Delft, Netherlands

**Keywords:** radiotherapy, glioma, fractional anisotropy, brain damage, diffusion tensor imaging (DTI), fluid attenuated inversion recovery (FLAIR)

## Abstract

**Background and purpose:**

To evaluate the value of diffusion tensor imaging (DTI) MRI derived fractional anisotropy (FA) and apparent diffusion coefficient (ADC) for both white matter (WM) and grey matter (GM) of the contralateral cerebrum following radiotherapy (RT) for supratentorial lower grade glioma (LGG) as markers for radiotherapy-induced brain damage (RIBD).

**Materials and methods:**

14 patients were analysed. WM and GM were segmented using automated software (cNeuro) and the mean FA and ADC were extracted per RT dose bin (0-10, 10-20, 20-30, 30-40, 40-50, >50 Gy) of WM and GM. One way ANOVA with *post-hoc* Bonferroni’s test were used to analyse differences in FA and ADC between dose bins. Fluid-attenuated inversion recovery (FLAIR) hyperintensities were segmented in a semi-automated manner and correlated with a percentual difference in ADC and FA between dose bin ≥50 Gy and the mean of lower dose bins. Furthermore, the correlation of raw values of these 3 metrics within dose bins was explored, and potential relations of changes to clinical parameters.

**Results:**

We observed changes in FA of WM for dose bin >50 Gy [(F(5, 74) = 5.461, p=0.0002)], but no changes in FA of GM and no changes in ADC for both WM and GM. The percentual change in ADC and FA in WM of dose bin >50 Gy did not correlate with the total volume of FLAIR hyperintensities of the contralateral cerebrum, and also the raw values of these metrics did not correlate within the >50 Gy dose bin, and only correlated with the Total Radiotherapy Dose delivered to the supratentorial brain.

**Conclusion:**

In the late phase after RT for LGG (average > 3 years), DTI-MRI derived FA values decreased significantly in WM in the cerebrum contralateral to the tumour, while no changes were observed in GM or in ADC values. The FA decrease is only observed in areas receiving the highest RT dose, allows for a localized assessment in the individual patient, and is not correlated with the observed total load of FLAIR hyperintensities within the contralateral cerebrum or changes in ADC, suggesting DTI-MRI and FLAIR derived metrics reflect RIBD in different ways.

## Highlights

On late follow-up MRI scans in patients treated with RT for LGG:

- DTI-MRI derived FA values decrease in WM in the healthy hemisphere.
- Significant FA decreases were observed in highest dose bins >50 Gy.
- Volume of FLAIR hyperintensities did not correlate to FA decrease
- FLAIR hyperintensities and FA decrease reflect RIBD in different ways

## Introduction

Radiotherapy-induced brain damage (RIBD) is a common problem in patients with lower grade glioma (LGG), which may result in delayed clinical symptoms, such as cognitive decline (1). Survival rates of patients with LGG are relatively high (median >10 years) with current state of the art therapies (2). Therefore, it is important to develop and establish radiological biomarkers detecting the underlying mechanisms of RIBD in this patient group.

Fluid-attenuated inversion recovery (FLAIR) hyperintensities and changes in diffusion have been readily linked to cognitive decline in patients with brain tumours (3). Diffusion tensor imaging (DTI) is often used in clinical practice in patients with brain tumours and has a potential to detect changes within white matter (WM) and grey matter (GM) that are linked to cognitive decline (4). However, it remains unclear how these changes are dependent on the delivered radiotherapy (RT) dose range and the time from the RT treatment completion. Changes in DTI-MRI derived metrics, including fractional anisotropy (FA) and mean diffusivity (MD) of WM, have readily been shown in patients with different grades of glioma (5–7). Notably, these studies showed a dependence of individual metrics on the used b-values for DTI-MRI, the tissue dose and scan timing, with generally late scans (approx. >12 months after end of RT) and areas with higher dose (approx. >50 Gy) showing significant changes. Interestingly, the sole study that evaluated DTI-MRI metric changes in GM did not observe any significant changes (6). In these aforementioned studies, WM was evaluated in both hemispheres, and with (in-house) research software packages to compute DTI-MRI derived metrics. This poses a need for a study minimizing (in)direct tumour effects on DTI-MRI metrics, involving clinical grade postprocessing methods and clinically routinely used DTI-MRI metrics, specifically apparent diffusion coefficient (ADC) and FA, to further clinical translation of findings and a better understanding of the inter-dependence of these metrics and potential added value and correlation of these metrics with FLAIR hyperintensities as observed on anatomical imaging.

In patients with brain tumours, it is challenging to determine whether the changes in radiological biomarkers are a result of RT alone or are caused by a combination of effects from RT, chemotherapy, surgery and local tumour infiltration - especially in case of high grade tumours and tumours not limited to a single hemisphere. In line with this, we hypothesize that in case of LGG limited to a single hemisphere, studying the healthy appearing cerebrum contralateral to the tumour could provide a potential way to more accurately assess RT effects (8, 9).

In this study we aim to explore the relationship of DTI-MRI derived ADC and FA changes in GM and WM with different RT dose bins in the otherwise “healthy” contralateral cerebrum in patients treated for LGG. For this purpose, we aim to use routine clinical FA and ADC maps output by the scanner as well as a clinically approved fully automated brain segmentation approach, augmented by a semi-automated FLAIR hyperintensity segmentation approach within the same workflow – this in order to facilitate optimal clinical translation of our findings. Finally, we aim to analyse the correlation of FLAIR hyperintensities in the contralateral cerebrum to the DTI-MRI derived metrics.

## Methods

### Study population

Patients (N=17) who received photon RT between 2007–2017 for LGG, WHO grade 2 and 3, at the radiotherapy department of the University Medical Center Groningen (UMCG), the Netherlands and were scheduled for regular follow-up visits were enrolled in a cross-sectional study in patients with LGG. For the current sub-study we included 14 of 17 patients of the LGG study who agreed to an extended DTI acquisition. A separate manuscript including the whole population and describing other MRI acquisitions was recently published (8). Inclusion criteria were: 1) >18 years old at the time of diagnosis, 2) ≥1 year after completion of RT, 3) absence of tumour progression requiring a new line of treatment. Patients were informed about the study prior to their next clinical follow-up appointment and were enrolled after providing written informed consent. The study was approved by the medical ethics board of the UMCG.

### MRI acquisition parameters

MRI scans (N=14) were acquired on a 3.0-Tesla Magnetom Prisma scanner (Siemens Healthineers, Erlangen, Germany – software version VE11C) with the following parameters. T1: voxel size 0.9x0.9x0.9 mm, slice thickness 0.9 mm, repetition time (TR) 2300 ms, echo time (TE) 2.32 ms, inversion time (TI) 900 ms, flip angle 8°, field-of-view (FOV) 230x230mm. FLAIR: voxel size 1.0x1.0x1.0 mm, TR 5000 ms, TE 391 ms, TI 1800 ms, flip angle 120°, FOV 230x230mm. DTI: voxel size 2.5x2.5x2.5 mm, slice thickness 2.5 mm, TR 2000 ms, TE 77.0 ms, FOV 220x220mm, using multi-directional diffusion weighted (MDDW) imaging in 64 directions and 3 b-values: 0, 1000, 2500 s/mm^2^ (number of averages 20, 1, 1, respectively).

### Image analysis

WM and GM were segmented using T1-weighted images using cNeuro cMRI (v1.11.0 (Combinostics Oy, Tampere, Finland, www.cneuro.com). FA and ADC maps were computed using the inline reconstruction pipeline of the MRI scanner using b-values 0 and 1000 s/mm^2^.

FLAIR images were processed for automatic segmentation and quantification of WM hyperintensity volume using the same cNeuro cMRI software suite (v1.11.0 (Combinostics Oy, Tampere, Finland, www.cneuro.com) with subsequent manual quality adjustments of the WM hyperintensity masks performed manually in ITK-SNAP v3.8.0 (10). The need for small manual adjustments was fully expected as we deployed the software outside of its primary intended use (being analysis of brain changes in dementia).

In order to account for the effects of surgery and tumour on the otherwise “healthy” brain tissue, the analysis was limited to the contralateral side of the cerebrum (using the tumour as a reference for the ipsilateral side).

**Figure 1:**
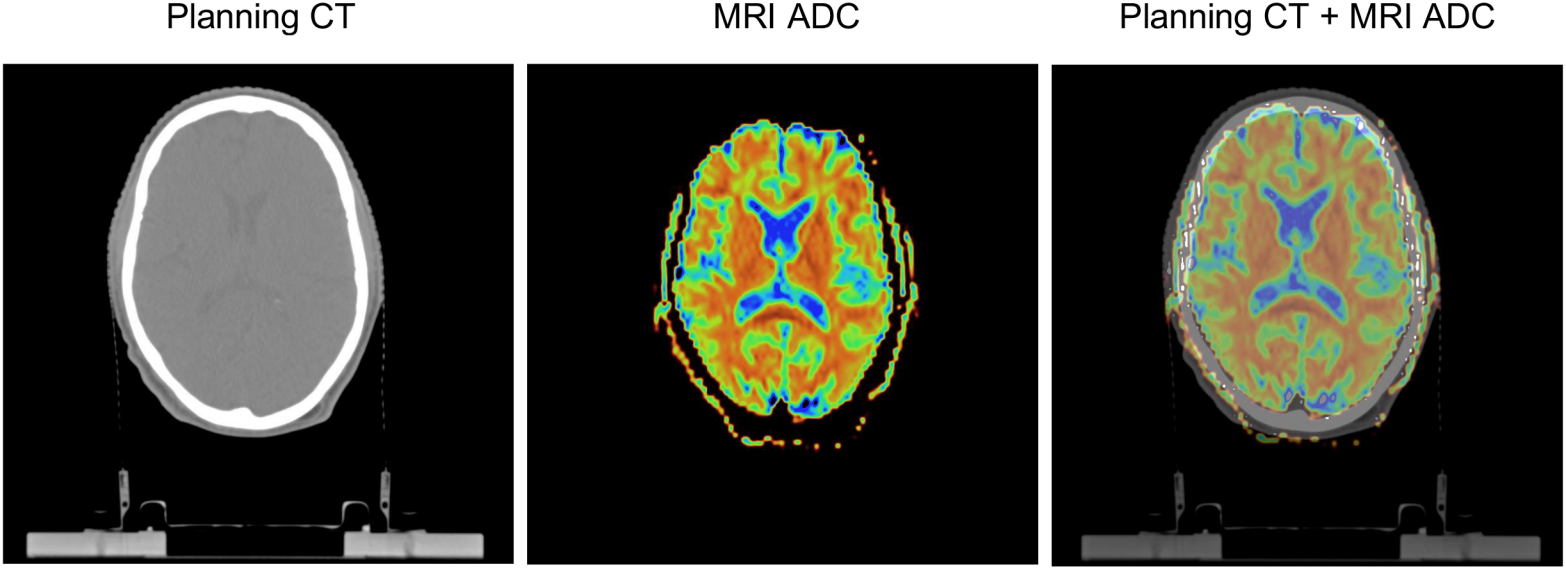
Rigid co-registration of MRI apparent diffusion coefficient (ADC) maps and planning CT. Images shown are obtained in a patient aged 51 years at RT onset, diagnosed with an oligodendroglioma in the right frontal lobe (not shown), and 46 months between the end of RT and the MRI scan.

**Figure 2:**
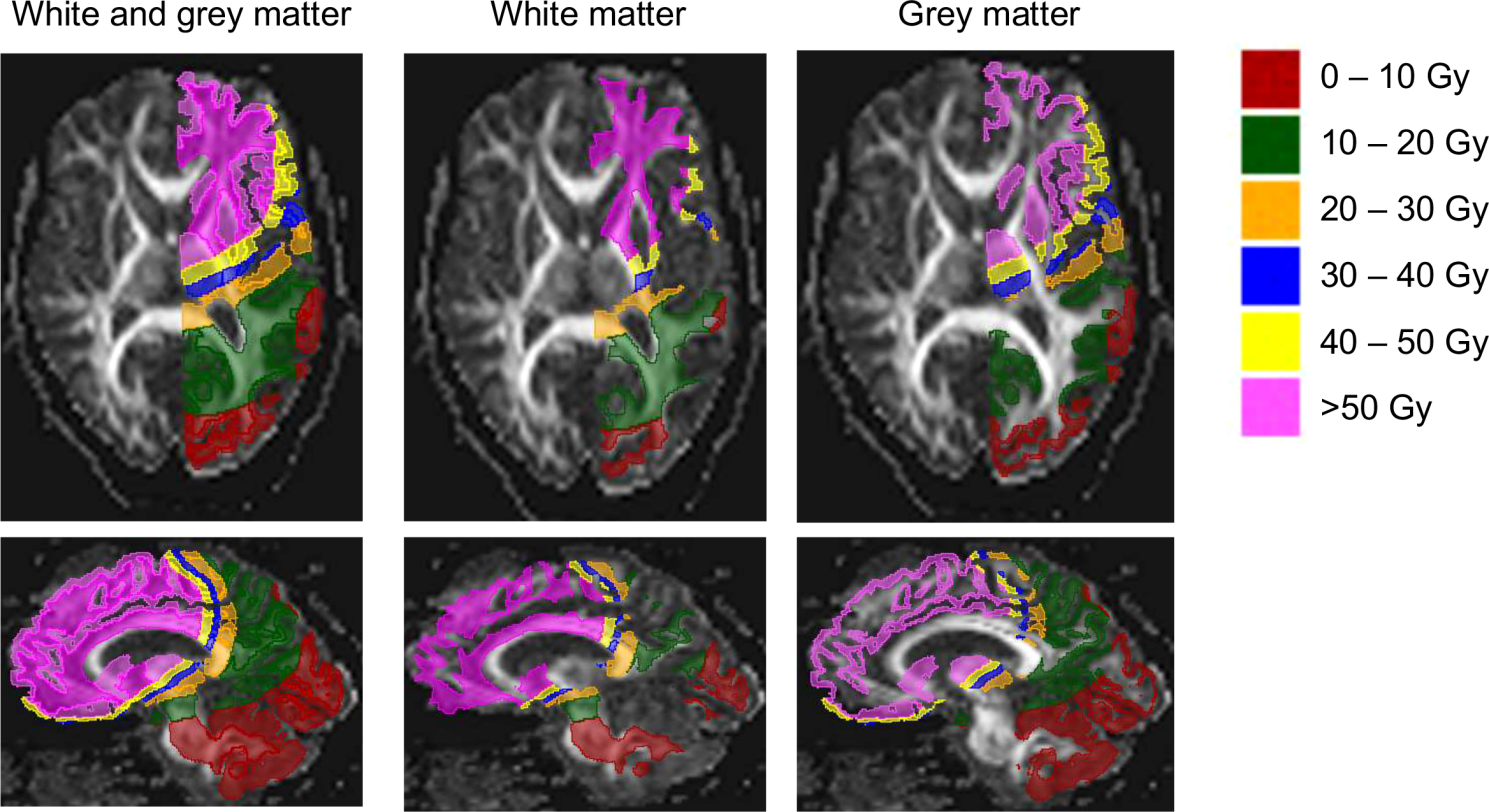
Segmentation shown for grey and white matter projected on fractional anisotropy (FA) maps. Images shown are obtained in a patient aged 51 years at RT onset, diagnosed with an oligodendroglioma in the right frontal lobe (not shown), and 46 months between the end of RT and the MRI scan.

All maps and masks were co-registered to RT planning-CT data using rigid co-registration (Elastix) in 3D Slicer (v4.11.20210226, https://www.slicer.org/). First, 6 masks based on different dose bins were generated by thresholding on the RT dose data, then each mask was intersected with a mask of the “healthy” hemisphere, and finally intersections were made with the WM, GM and FLAIR hyperintensities masks. For an example see **Figure 1**. In order to analyse the mean FA and ADC of WM and GM, the RT dose maps differentiated 6 dose bins: 0-10, 10-20, 20-30, 30-40, 40–50 and >50 Gy (**Figure 2**).

### Statistical analysis

Descriptive statistics were used for evaluating the clinical characteristics of the study population. The data was inspected for normality with D’Agostino & Pearson, Anderson-Darling, Shapiro-Wilk and Kolmogorov-Smirnov tests. Accordingly, One-way ANOVA test was used for evaluating the differences in ADC and FA between different dose bins for both WM and GM and Bonferroni’s correction was applied as a *post-hoc* multiple comparisons test. The Kruskal-Wallis test was used to compare the volume of FLAIR hyperintensities between dose bins, as FLAIR hyperintensities were not normally distributed.

Spearman’s correlation was used to test the relationship between the percentual change in FA of the highest dose bin with the mean of lower dose bins and the volume of FLAIR hyperintensities, as well as for the correlation of FLAIR hyperintensities with the raw FA and ADC values within each individual dose bin. Spearman’s correlation was also used to explore the relation of continuous clinical variables (Age, Tumour size etc.) and their relationship to changes in WM ADC, WM FA and the volume of FLAIR hyperintensities.

Multiple Linear Regression was used to study the relationship of individual categorical clinical parameters (RT Technique, Chemotherapy etc.) individually - because of the limited number of subjects, it was not possible to perform Multiple Linear Regression for all the features at once.

The significance level was set at a two-tailed p value of 0.05. Statistical analysis was performed using GraphPad Prism version 9.5.1 for macOS, GraphPad Software, San Diego, California USA, www.graphpad.com.

## Results

The population included 8 males and 6 females. In these patients the LGG tumour was located in the temporal (42.9%), frontal (28.6%), insular (21.4%) and parietal (7.1%) lobe. The tumour pathological subtype consisted of astrocytoma grade 2 (57.2%), oligodendroglioma grade 2 (35.7%) and oligodendroglioma grade 3 (7.1%). One oligodendroglioma grade 2 was IDH1 negative, one oligodendroglioma grade 2 not tested, and the remaining tumours were IDH1 mutated.

The mean patient age at the time of RT onset was 42.6 years (26–58 yrs.) and the mean time interval between the end of RT treatment and the date of the MRI scan performed in the context of the study was 39.1 months. The mean planning target volume (PTV) and clinical target volume (CTV) were 286.4cc and 194.7cc, respectively. The mean prescribed photon RT dose was 52.6 Gy. Radiotherapy treatment planning techniques included 3D CRT (3 Dimensional Conformal RT) (7.1%), IMRT/VMAT (Intensity Modulated RT/Volumetric Modulated Arc Therapy) (85.7%) and fractionated SRT (Stereotactic RT) (7.1%).

Twelve patients (85.7%) received chemotherapy with PCV (Procarbizine, Lomustine, Vincristine) sequential to RT, including one patient who also received TMZ (Temozolomide) prior to RT. Most patients underwent only a single tumour surgery (64.3%), 4 underwent multiple surgeries (28.6%) and one patient underwent a biopsy only. Detailed patient’s characteristics are provided in **Table 1**.

**Table 1:** Clinical characteristics of patients.

Patient Age	Median (IQR)
Age at RT onset	41.50 (37.75-51.00)
Age at the time of a scan	45.50 (40.50-54.25)
Clinical characteristics	number (%)
Hypertension	none
Diabetes	2 (14.3)
Epilepsy	12 (85.7)
Smoking	8 (57.1)
Tumour type	number (%)
Astrocytoma IDH1 mutated, WHO grade 2	8 (57.2)
Oligodendroglioma, WHO grade 2	5 (35.7)
Oligodendroglioma, WHO grade 3	1 (7.1)
Tumour location	number (%)
Frontal	4 (28.6)
Temporal	6 (42.9)
Insular	3 (21.4)
Parietal	1 (7.1)
Chemotherapy	number (%)
None	2 (14.3)
PCV sequential to RT	11 (78.6)
TMZ prior to RT and PCV sequential to RT	1 (7.1)
Surgery	number (%)
Biopsy only	1 (7.1)
Single	9 (64.3)
Multiple	4 (28.6)
RT type	number (%)
3D CRT	1 (7.1)
IMRT/VMAT	12 (85.7)
Fractionated SRT	1 (7.1)
RT treatment	mean (SD)
Tumour size – largest tumour diameter [cm]	5.82 (2.04)
Prescribed RT dose [Gy]	52.59 (2.658)
Mean CTV [cc]	194.7 (99.33)
Mean PTV [cc]	286.4 (114.3)
Age at RT	42.6 (8.75)
Age at the time of a scan	46.0 (8.98)
RT to scan interval	39.1 (17.96)
RT mean dose [Gy]	mean (SD)
Cerebrum	25.67 (6.900)
CL cerebrum	16.39 (7.329)

WHO, World Health Organization; IDH1, isocitrate dehydrogenase 1 mutation; RT, radiotherapy; TMZ, temozolomide; RT, radiotherapy; PCV, procarbazine, lomustine and vincristine; 3D CRT, three-dimensional conformal radiation therapy; VMAT, volumetric modulated arc therapy; IMRT, intensity-modulated radiation therapy; SRT, stereotactic radiotherapy; CTV, clinical target volume; PTV, planning target volume; CL, contralateral.

**Figure 3:**
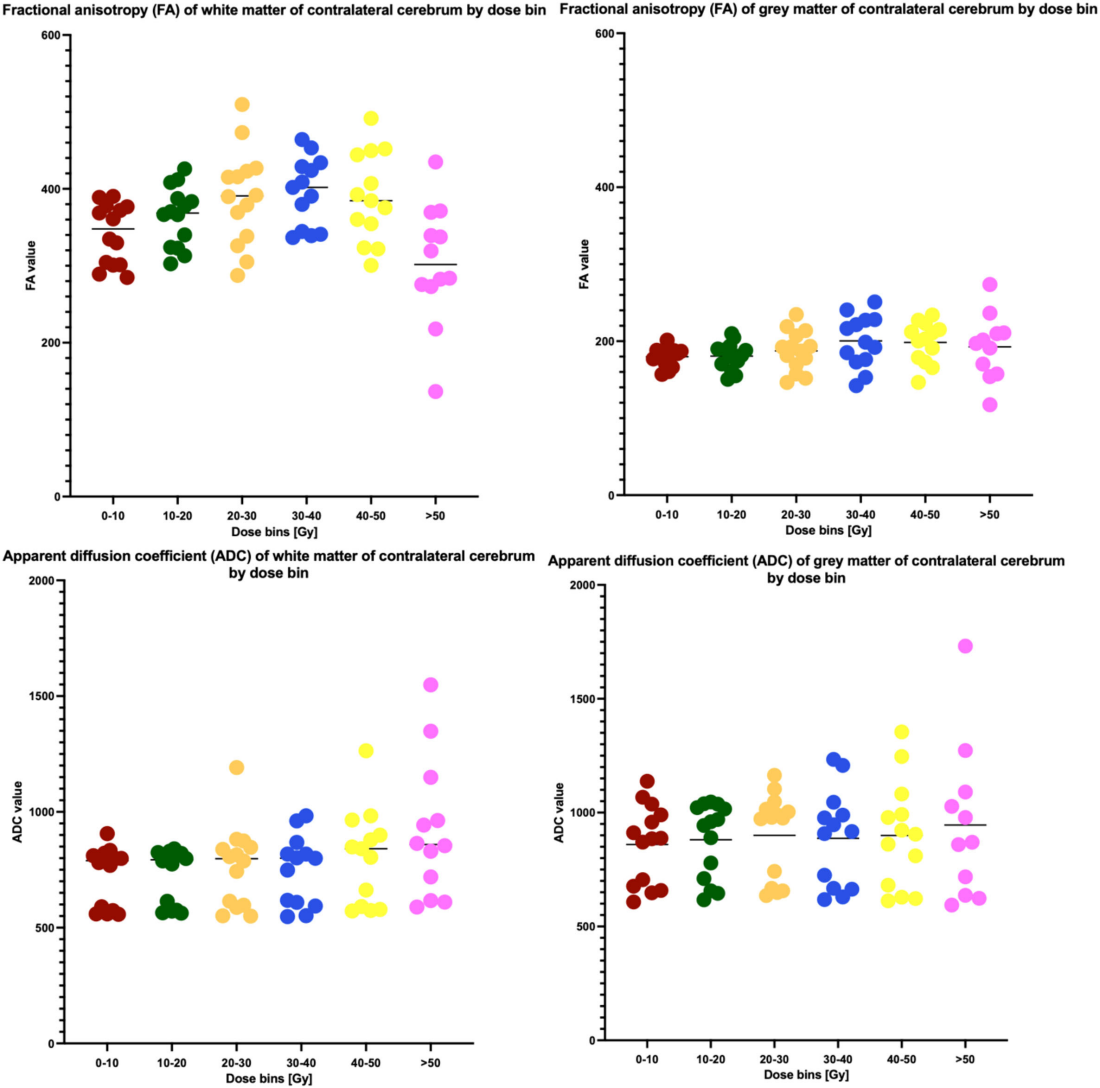
Fractional anisotropy (FA) and apparent diffusion coefficient (ADC) values in white matter and grey matter in contralateral cerebrum by dose bin.

We identified a statistically significant difference in the mean FA of WM of the contralateral cerebrum between at least two dose bins (F(5, 74) = 5.461, p=0.0002). Bonferroni’s multiple comparisons test showed that the mean value of FA of WM of the contralateral cerebrum of dose bin >50 Gy was significantly different from dose bins 20–30 Gy (p=0.0022, 95% C.I. = 20.72, 151.0), 30–40 Gy (p=0.0010, 95% C.I. = 26.13, 158.7) and 40–50 Gy (p=0.0030, 95% C.I. = 19.29, 151.9). There were no statistically significant differences in the mean ADC of WM in the contralateral cerebrum between dose bins (F(5, 74) = 1.963, p=0.0941). For GM of the contralateral cerebrum, no significant difference was found between dose bins for the mean value of FA, as well as for the mean value of ADC (**Figure 3**). Also, the volume of FLAIR hyperintensities did not significantly differ across all dose bins (p=0.5646).

Next, we correlated the changes in ADC and FA in the >50 Gy dose bin with FLAIR hyperintensity volume in the same contralateral hemisphere. In order to achieve this, we first calculated the percentual change of ADC and FA in the >50 Gy dose bin compared to the respective mean of ADC or FA values of all preceding dose bins, and then performed the correlation of this change with FLAIR hyperintensity volume. However, no statistically significant correlation with FLAIR hyperintensities volume was observed for changes in either ADC or FA (r=-0.343 and p=0.2725, r=0.077 and p=0.8124, respectively).

In addition to this, we explored the correlations of FLAIR hyperintensities with the raw FA and ADC values in WM per dose bin, *for all dose bins*. The only significant correlations observed were FLAIR hyperintensities vs. FA in dose bin 30–40 Gy (Spearman r =-0.6045, p=0.0319) and FLAIR hyperintensities vs. ADC in dose bin 10–20 Gy (Spearman r =0.5673, p=0.0373) – thus no significant correlation was found for these 3 metrics in the dose bin >50 Gy with the aforementioned significantly lower FA values in WM.

Finally, we explored if clinical parameters were related to changes observed in ADC, FA and the total volume of FLAIR hyperintensities. As a first step the following *clinical categorical variables* were included in the analysis: Radiotherapy Technique, Chemotherapy, Tumour Type/Grade, Smoking, Diabetes, Epilepsy, Number of Resections and Tumour Location. However, no significant relationships were observed. As a second step, we included the following *continuous variables* in the analysis: Age, Tumour Size, Total Radiotherapy Dose to the supratentorial brain, and Total Radiotherapy Dose to the supratentorial brain contralateral to the tumour. This analysis only showed a statistically significant correlation of changes in WM ADC (r = -0.7063, p = 0.0129), WM FA (r = 0.7762, p = 0.0043) and the total volume of FLAIR hyperintensities (r = 0.5560, p = 0.0419) with the Total Radiotherapy Dose to the supratentorial brain, and thus not with any of the other aforementioned variables.

## Discussion

To our knowledge, this is the first study to evaluate DTI-MRI derived ADC and FA values for both WM and GM in different RT dose bins, specifically in the contralateral cerebrum of patients treated with RT for supratentorial LGG. In this study performed using 3.0T MRI, deploying routinely available ADC and FA maps and clinical brain structure segmentation routines - the changes in FA were observed in WM in the areas of dose bin >50 Gy, but interestingly not in lower dose bins. Furthermore, no changes were observed in FA of GM and no changes in ADC of both WM and GM. We also showed that the percentual change in FA of dose bin >50 Gy did not correlate with the total volume of FLAIR hyperintensities of the contralateral cerebrum.

This study is in line with previous studies showing that DTI-MRI is able to show RIBD in WM of patients treated with RT for glial tumours (5–7, 11), with the common denominator of these studies being that DTI-MRI derived metrics can observe changes after radiotherapy vs. baseline beyond 12 months and that non-routine clinical markers like MD and especially radial diffusivity (RD) seem to outperform FA with regard to being able to detect significant changes in areas having received 10–20 Gy and up and 20–30 Gy and up, respectively. Of note is that even in the study displaying some of the lowest detection limits for DTI-MRI derived markers, *maximum observed changes* are fairly modest, being 6% relative change for FA and 10 for RD (at 18 months after RT, dose >50 Gy) (6). A single study on a population with LGG reported an isolated decrease in FA at 45–50 Gy (7). These differences may stem from the different grade of the tumours and the level of tumour infiltrations into the brain tissue, as well as the extent of surgery needed to debulk/resect the tumour and related RT dose distribution. Other factors having a potentially significant influence could be vendor-related DTI acquisition differences – including b-values and MRI field strength used - but also the timepoint at which the scans were acquired. Unlike the studies listed above where observational period was no longer than 2 years, our study has a longer time interval between RT and the MRI scan, which was on average 3 years. Interestingly, Haris et al. reported partial recovery of FA of WM at 14 months from RT (7). Thus, it may be that the observed decrease in FA of WM restricted to the areas of dose bins >50 Gy in our study is reflecting partially recovered tissue in areas with a lower dose. Alternatively, our observations could be a result of exclusively including the contralateral cerebrum with RT dose bath which avoids including scar tissue after surgery and may include fewer local infiltrations from the potentially remaining tumour tissue. However, the findings of this study indicate that RT dose bath in the contralateral cerebrum, where the influence of tumour growth and surgery is expected to be much lower than in the ipsilateral cerebrum, still has an impact on radiological markers of RIBD on follow-up MRI images. This finding is especially relevant in the population of patients suffering from LGGs, which are often restricted to one side of the cerebrum.

Our study did not show any changes in FA of GM, similarly to the findings of Raschke et al. (6), which could suggest that GM is less vulnerable to RT, or that the DTI-derived FA is not a sensitive enough marker for assessment of RIBD in GM, especially in the areas distant to the tumour receiving lower RT dose. Similarly, we did not observe any significant changes in ADC of WM and GM in our study population. The results of this study suggest that FA is a better late indicator of RIBD than ADC, particularly in follow-up scans in the late-delayed phase after RT – in our case around 3 years after the end of RT. These results are in line with previous literature suggesting that ADC is primarily a marker of a recurrent tumour rather than RIBD (12).

Interestingly, the percentual change in FA of dose bin >50 Gy, thus the only area where a change in FA was observed in comparison to other dose bins, did not correlate with the total volume of FLAIR hyperintensities of the contralateral cerebrum, although this would be expected as FLAIR and DTI are both MRI sequences used in visualizing WM changes. However, this finding is in line with a previous study by Harris et al. where changes in FA were observed although no WM changes were visible yet on FLAIR images (7). In line with this, a study by Castellano et al. highlighted the temporal and spatial discordance of DTI derived metrics and FLAIR hyperintensities in patients with LGG during chemotherapy (13). Moreover, this finding further suggests that looking only at a single radiological imaging biomarker of RIBD may not deliver enough information about the actual damage to the brain tissue. Therefore, patients should be scanned with multiple sequences which should be analysed in the light of the timepoint after RT at which they were obtained. Interestingly, the percentual changes in both FA, ADC and total volume of FLAIR hyperintensities did correlate with the total dose delivered to the supratentorial brain – although from a clinical practical perspective a global correlation does not provide a lot of insight in the individual patient due to the lack of spatial information, in contrast to the knowledge which can be obtained regarding the presence (or absence) of lower FA values in the dose bin >50 Gy in the contralateral hemisphere and brain structures contained within this dose area in the individual patient.

The limitations of the study are a small study population with various RT treatment planning techniques, adjuvant chemotherapies and tumour locations within the population. Furthermore, no pre-RT baseline scan was available to compare the studied metrics to in the context of our cross-sectional design. However, such a diversity is a very common issue in studies concerning relatively rare brain tumours because heterogeneity is also a clinical challenge.

In conclusion, in the late phase after RT treatment for LGG, DTI-MRI derived FA values, but not ADC values decrease in WM, but not GM in the cerebrum contralateral to the location of the tumour. Furthermore, this decrease is only observed in areas receiving the RT dose >50 Gy and does not seem to be correlated with the observed total load of FLAIR hyperintensities within the same side of the cerebrum, suggesting that DTI-MRI and FLAIR derived metrics likely reflect RIBD in different ways.

LGG patients should be scanned with multiple MRI sequences which each should be analysed and interpreted in the light of the timepoint after RT at which they were obtained, as well as the approximate dose delivered to the areas where the observations are made.

## Data Availability

The original contributions presented in the study are included in the article/supplementary material. Further inquiries can be directed to the corresponding author.

## Glossary

RIBD: Radiotherapy-induced brain damage
LGG: Lower grade glioma
FLAIR: Fluid-attenuated inversion recovery
DTI: Diffusion tensor imaging
WM: White matter
GM: Grey matter
RT: Radiotherapy
FA: Fractional anisotropy
MD: Mean diffusivity
ADC: Apparent diffusion coefficient
WHO: World Health Organisation
UMCG: University Medical Center Groningen
TR: Repetition time
TE: Echo time
TI: Inversion time
FOV: Field-of-view
MDDW: Multi-directional diffusion weighted
PTV: Planning target volume
3D CRT: Three-dimensional conformal radiation therapy
IMRT: Intensity-modulated radiation therapy
VMAT: Volumetric modulated arc therapy
SRT: Stereotactic radiotherapy
PCV: Procarbazine, lomustine and vincristine
TMZ: Temozolomide.
